# Tripartite motif-containing 3 (TRIM3) inhibits tumor growth and metastasis of liver cancer

**DOI:** 10.1186/s40880-017-0240-5

**Published:** 2017-09-26

**Authors:** Xu-Qiong Huang, Xiao-Fei Zhang, Jin-Hua Xia, Jie Chao, Qiu-Zhong Pan, Jing-Jing Zhao, Zi-Qi Zhou, Chang-Long Chen, Yan Tang, De-Sheng Weng, Jian-Hua Zhang, Jian-Chuan Xia

**Affiliations:** 1 Department of Biotherapy, State Key Laboratory of Oncology in South China, Collaborative Innovation Center for Cancer Medicine, Sun Yat-sen University Cancer Center, Guangzhou, 510060 Guangdong P. R. China; 2 Department of Epidemiology and Health Statistics, Guangdong Pharmaceutical University, Guangzhou, 510010 Guangdong P. R. China; 3 Guangzhou Health Science College, Guangzhou, 510925 Guangdong P. R. China; 4 Shanxi Entry-Exit Inspection and Quarantine Bureau, Xi’an, 710068 Shanxi P. R. China

**Keywords:** Tripartite motif-containing 3 (*TRIM3*), Liver cancer, Tumor suppressor, Cell cycle

## Abstract

**Background:**

Reduced expression of tripartite motif-containing 3 (*TRIM3*) has been reported to be involved in the pathogenesis of human glioblastoma. In our previous research, we found that *TRIM3* expression was markedly reduced in human primary hepatocellular carcinoma (HCC) tissues and that low *TRIM3* expression was associated with short survival of HCC patients. However, the role of TRIM3 in liver cancer remains unknown. This study aimed to investigate the function of TRIM3 in liver cancer cells.

**Methods:**

The protein levels of TRIM3 in five liver cancer cell lines (SK-Hep1, Hep3B, Huh7, HepG2, Bel-7402) and one normal liver cell line (L02) were detected with Western blotting. HepG2 and Bel-7402 cells with low TRIM3 expression were infected with recombinant lentiviruses overexpressing TRIM3 (LV-TRIM3), whereas Huh7 and Hep3B cells with high TRIM3 expression were transfected with TRIM3-targeted small interfering RNA (siTRIM3). The functions of TRIM3 in the proliferation, colony formation, cell cycle, migration, invasion, and apoptosis of the above cell lines were examined. The effect of TRIM3 on tumor growth and metastases in nude mice was also investigated.

**Results:**

TRIM3 was overexpressed in HepG2 and Bel-7402 cells with LV-TRIM3 infection, which further reduced proliferation, colony formation, migration, and invasion of both cell lines. Cell cycle analysis showed that TRIM3 overexpression induced G_0_/G_1_ phase arrest in HepG2 and Bel-7402 cells. Moreover, apoptosis was not increased in HepG2 or Bel-7402 cells overexpressing TRIM3. Contrarily, silencing TRIM3 expression in Huh7 and Hep3B cells by siTRIM3 led to significantly decreased percentages of both cells in the G_0_/G_1_ phase and promoted cell proliferation, colony formation, migration, and invasion. In vivo experiment results confirmed that TRIM3 overexpression suppressed tumor growth and metastasis.

**Conclusions:**

TRIM3 plays a tumor-suppressing role in the regulation of liver cancer development by reducing cell proliferation through cell cycle arrest at the G_0_/G_1_ phase.

## Introduction

The most common histologic type of liver cancer is hepatocellular carcinoma (HCC) which is the sixth most prevalent cancer and the third most frequent cause of cancer-related death [1]. About 750,000 new cases of HCC are diagnosed and nearly 700,000 HCC-related deaths occur each year [2]. Most HCC cases were reported in developing countries in Asia and Africa, especially where the main risk factor is hepatitis B virus infection [3]. Currently, surgical resection, radiofrequency ablation, and liver transplantation are considered potentially curative therapies and provide the possibility of long-term remission in patients with early-stage HCC [4–6]. However, the intrahepatic recurrence rate remains high [7], and recurrence in advanced HCC patients after treatments cannot always be prevented [8, 9]. Recently, a molecular study [10] has showed that some somatic mutations, for example, *TP53* mutation, were associated with the development and progression of HCC. Understanding these alterations and underlying molecular mechanisms will be critical for the improvement of diagnosis, treatment, and prognostic prediction of HCC.

Increasing clinical evidence shows that the deregulation of ubiquitin-mediated degradation of tumor suppressors or oncogene products is likely to be involved in the development and progression of carcinomas [11]. Ubiquitin conjugation is catalyzed by ubiquitin-activating enzyme (E1), ubiquitin-conjugating enzyme (E2), and ubiquitin ligase (E3). E3 is a scaffold protein that mediates ligation between E2 and the substrate; it is thought to be the component that recognizes the substrate most directly. Based on processing of covalent linkage with ubiquitin, E3 enzymes have been classified into two families: the HECT (homologous to the E6-AP carboxyl terminus) family and the RING (really interesting new gene) family. Tripartite motif (TRIM) proteins constitute a subfamily of the RING-type E3 family. Almost all TRIM proteins have a RING domain, one or two B-box domains, and a coiled-coil domain [12, 13]. Several types of TRIM proteins mediate protein degradation via their RING domains [14–18]. Several *TRIM* family genes—including *TRIM11*, *TRIM13*, *TRIM24*, and *TRIM28*—are found to be altered in cancers or cancer-related diseases [19–22]. These alterations include deletion, translocation, and amplification, which demonstrate the functional diversity of TRIM proteins in cancers.

The human *TRIM3* gene is localized at chromosome 11p15.5, a region that has been found to contain numerous cancer-related genes among multiple cancers [23, 24]. This observation indicates that the *TRIM3* may be a novel tumor-related gene. Our previous study indicated that *TRIM3* expression was down-regulated in HCC at both the mRNA and protein levels and that low *TRIM3* expression was associated with an unfavorable prognosis [25]. To elucidate the potential role of TRIM3 in the development of liver cancer, we investigated the functions of TRIM3 in liver cancer cell lines.

## Materials and methods

### Cell lines and culture conditions

Human liver cancer cell lines HepG2, Hep3B, and SK-Hep1 were obtained from the American Type Culture Collection (ATCC, Manassas, VA, USA). The HCC cell line Huh7 was obtained from the RIKEN cell bank (Ibaraki, Osaka, Japan). The HCC cell line Bel-7402 and normal liver cell line L02 were obtained from the Committee of Type Culture Collection of the Chinese Academy of Sciences (CTCCCAS, Shanghai, China). All cells were cultured in 5% CO_2_ at 37 °C in RPMI-1640 (Gibco, Grand Island, NY, USA), supplemented with 10% fetal bovine serum (FBS, Gibco) and 1% penicillin–streptomycin (Invitrogen, Grand Island, NY, USA).

### Protein extraction and Western blotting

Total protein was extracted from cells using Radio-Immunoprecipitation Assay (RIPA) Lysis Buffer (Beyotime, Shanghai, China). The concentration of total protein was measured with a Bicinchoninic Acid Protein Assay Kit (BioRad, Hercules, CA, USA). Equal quantities (30 μg) of proteins underwent electrophoresis in 12% sodium dodecyl sulfate–polyacrylamide gels, and then the proteins in gels were transferred onto polyvinylidene difluoride membranes (BioRad). After being blocked in 8% non-fat milk in phosphate-buffered saline-Tween (PBST) for 1 h, the membranes were incubated with primary rabbit anti-TRIM3 polyclonal antibody (1:500 dilution, Abcam, Cambridge, MA, USA) and rabbit anti-glyceraldehyde-3-phosphate dehydrogenase (GAPDH) polyclonal antibody (1:10,000 dilution, Proteintech, Chicago, IL, USA) at 4 °C overnight. Afterwards, the membranes were washed and incubated with horseradish peroxidase-conjugated goat anti-rabbit antibody (1:5000 dilution, Cell Signaling Technology, Danvers, MA, USA) at room temperature for 1 h, followed by three washes with PBST. Band intensity was measured by densitometry using Quantity One software (BioRad). Expression levels of TRIM3 protein were normalized to that of GAPDH.

### Cell transfection with recombinant lentivirus and small interfering RNA (siRNA)

Recombinant lentiviruses expressing TRIM3 (LV-TRIM3) and negative control vector (LV-NC) were obtained from GenePharma (Shanghai, China). Multiplicity of infection (MOI) refers to the number of virions that are added per cell during infection (e.g., the MOI is 50 when 50 virions are added to one cell). HepG2 and Bel-7402 cells were seeded separately in 6-well plates at a density of 2 × 10^5^ cells per well and cultured until they reached 80%–90% confluence. Then the above cells were infected with LV-NC at MOI of 50 and LV-TRIM3 at MOIs of 50, 100, 200, and 400 by adding gradient volume of virus solution into the culture medium in the presence of 5 mg/mL polybrene (Sigma-Aldrich, St. Louis, MO, USA), respectively. After 48-h infection, the cells were selected with 2 mg/mL puromycin (Biosharp, Shanghai, China) for 14 days, and puromycin-resistant cells were pooled and cultured for further analysis. The selection criteria for MOI were based on the level of TRIM3 expression in HepG2 and Bel-7402 cells and the corresponding number of virions used, namely, considerably high TRIM3 expression level with as few virions to be used as possible. The selected stable cell lines infected with indicated recombinant lentiviruses were designated as HepG2/TRIM3, HepG2/NC, Bel-7402/TRIM3, and Bel-7402/NC, respectively, and used for subsequent assays.

To silence the expression of TRIM3, three TRIM3-targeted siRNAs (siTRIM3 #1–3) and a negative control siRNA (siNC) synthesized by GenePharma were used. The siRNA sequences are shown in Table 1. Huh7 and Hep3B cells were transiently transfected with siRNAs using Lipofectamine RNAi Max Reagent (Invitrogen) according to the manufacturer’s instructions. Briefly, Huh7 and Hep3B cells were seeded separately in 6-well plates at a density of 2 × 10^5^ cells per well and cultured until they reached 80%–90% confluence. Then, the above cells were transfected with siRNAs in serum- and antibiotic-free medium for 8 h. Afterwards, the medium was replaced, and the cells were cultured for 48 h prior to colony formation, cell proliferation, and cell cycle analyses. The transfected cells were designated as Huh7/siTRIM3, Huh7/siNC, Hep3B/siTRIM3, and Hep3B/siNC, respectively.

**Table 1:** Small interfering RNA (siRNA) sequences that target tripartite motif-containing 3 (siTRIM3) and negative control siRNA (siNC)

siRNA	Sense sequence	Anti-sense sequence
siTRIM3 #1	5′-GCUCACUGUCACUACCAAATT-3′	5′-UUUGGUAGUGACAGUGAGCTT-3′
siTRIM3 #2	5′-GGAAGGGAGAAAGGUGAAUTT-3′	5′-AUUCACCUUUCUCCCUUCCTT-3′
siTRIM3 #3	5′-GCUGGCAACCACUGCUUUATT-3′	5′-UAAAGCAGUGGUUGCCAGCTT-3′
siNC	5′-UUCUCCGAACGUGUCACGUTT-3′	5′-ACGUGACACGUUCGGAGAATT-3′

### Proliferation assay

Cell proliferation was measured with the colorimetric assay using the methanethiosulfonate (MTS) reagent (Sigma-Aldrich). Cells were seeded in 96-well plates (800 cells/well) and cultured for 1–7 days. Afterwards, 20 μL of MTS reagent (5 mg/mL) was added to each well, and cells were incubated for 3 h in 5% CO_2_ at 37 °C to quantify the optical density (OD) at 490 nm. Each kind of cells was measured in triplicate wells, and independent experiments were repeated three times.

### Colony formation assay

Cells were seeded in 6-well plates (500 cells/well) and cultured at 37 °C in an atmosphere of 5% CO_2_ for 12 days. Each measurement was performed in triplicate wells. Cell colonies were washed twice with 1× PBS (Gibco), fixed in 75% alcohol for 30 min, and stained with 0.5% crystal violet (Beyotime) for 30 min. Colonies that contained more than 50 cells were counted. Colony-forming efficiency (CFE) was calculated using the formula: CFE = colony number/plated cell number × 100%.

### Cell cycle analysis

The cell cycle was analyzed with flow cytometry (Beckman Coulter, Fullerton, CA, USA). Briefly, cells were harvested and washed twice with 1× PBS and fixed in 75% ethanol at −20 °C overnight. Then, the cells were washed twice and re-suspended in ice-cold 1× PBS containing 0.1 mg/mL RNAse (Sigma-Aldrich). After being incubated at 37 °C for 30 min, the cells were re-suspended in ice-cold 1× PBS containing 50 mg/mL propidium iodide (PI; Bestbio, Shanghai, China) at 4 °C in the dark. DNA content was then analyzed on a flow cytometer (Beckman Coulter, Fullerton, CA, USA). The distribution of cells at each cell cycle phase was calculated using Cell Quest and ModFit software (Beckman Coulter).

### Apoptosis assay

Apoptosis was analyzed with the Annexin V-fluorescein isothiocyanate (FITC) plus PI staining kit (BD Biosciences, Bedford, MA, USA) using flow cytometry according to manufacturer’s instructions. Cells were collected and centrifuged at 1200 r/min for 5 min). After washing twice with cold 1× PBS, the cells were re-suspended in 400 μL of 1× binding buffer, then incubated with 5 μL Annexin V-FITC and 5 μL PI in the dark at 4 °C for 15 min. The stained cells were analyzed with flow cytometry. The measurements were performed in triplicate.

### Transwell migration and invasion assays

Cell migration and invasion assays were performed using inserts with 8-μm pore-size polycarbonate membrane (Corning, Corning, NY, USA) placed in matched 24-well cell culture plates. For the migration assay, cells (2 × 10^5^) in 200 μL RPMI-1640 were seeded into the upper chamber without Matrigel; for the invasion assay, cells (3 × 10^5^) in 200 μL RPMI-1640 were seeded into the upper chamber where membranes were pre-coated with a thin layer of 0.5 mg/L Matrigel (BD Biosciences). For both assays, 500 μL RPMI-1640 containing 10% FBS was added to the lower chambers. The plates were incubated for 24 h (migration) or 48 h (invasion), then cells that had migrated or invaded to the bottom of the membrane were fixed with 75% methanol for 30 min and stained with 0.5% crystal violet for 60 min. Cells were counted in 10 random fields for each membrane under a microscope (Nikon, Fukok, Japan) at 200× magnification. Representative fields were photographed under the microscope at 100× magnification. Each measurement was carried out in triplicate wells, and independent experiments were repeated three times.

### In vivo experiments

Four- to 5-week-old female BALB/c nude mice were purchased from the Shanghai Laboratory Animal Company (SLAC, Shanghai, China). Tumor growth assays were performed as previously described [26]. Briefly, for each cell line, 5 × 10^6^ cells in 100 μL PBS were injected subcutaneously into the posterior flanks of mice, with 5 mice per group. The width and length of the formed tumors were measured every 3 days. The tumor volume was calculated using the following formula: tumor volume  =  width^2^ × length / 2. After approximately 6 weeks, the mice were killed by cervical dislocation, and the tumors were harvested to measure the volume and weight. Proteins were extracted from tumor tissues for Western blotting as previously described.

For in vivo metastasis assay, mice were injected with 2 × 10^6^ cells in 100 μL PBS through the lateral tail vein, with seven mice per group. At 7–8 weeks after inoculation, all mice were killed by cervical dislocation, and the lungs were harvested and fixed in 4% paraformaldehyde. Subsequently, the lungs were embedded in paraffin, and serial 2-μm-thick sections of whole lungs were obtained and examined, using hematoxylin and eosin (H&E) staining to identify the metastases.

Experiments were approved by the Laboratory Animal Ethics Committee of Sun Yat-sen University (Guangzhou, Guangdong, China). All experimental procedures involving animals were performed in accordance with the Guide for the Care and Use of Laboratory Animals (National Institutes of Health publication Nos. 80-23, revised in 1996) and the institutional ethical guidelines for animal experiments.

### Statistical analysis

All statistical analyses were performed using SPSS version 21 software (IBM, Chicago, IL, USA). A two-tailed unpaired Student’s t test was used for statistical comparisons between two groups. Two-sided *P* values less than 0.05 were considered statistically significant. Where appropriate, data are presented as mean  ± standard deviation.

## Results

### Expression of TRIM3 in liver cancer cell lines

**Fig. 1:**
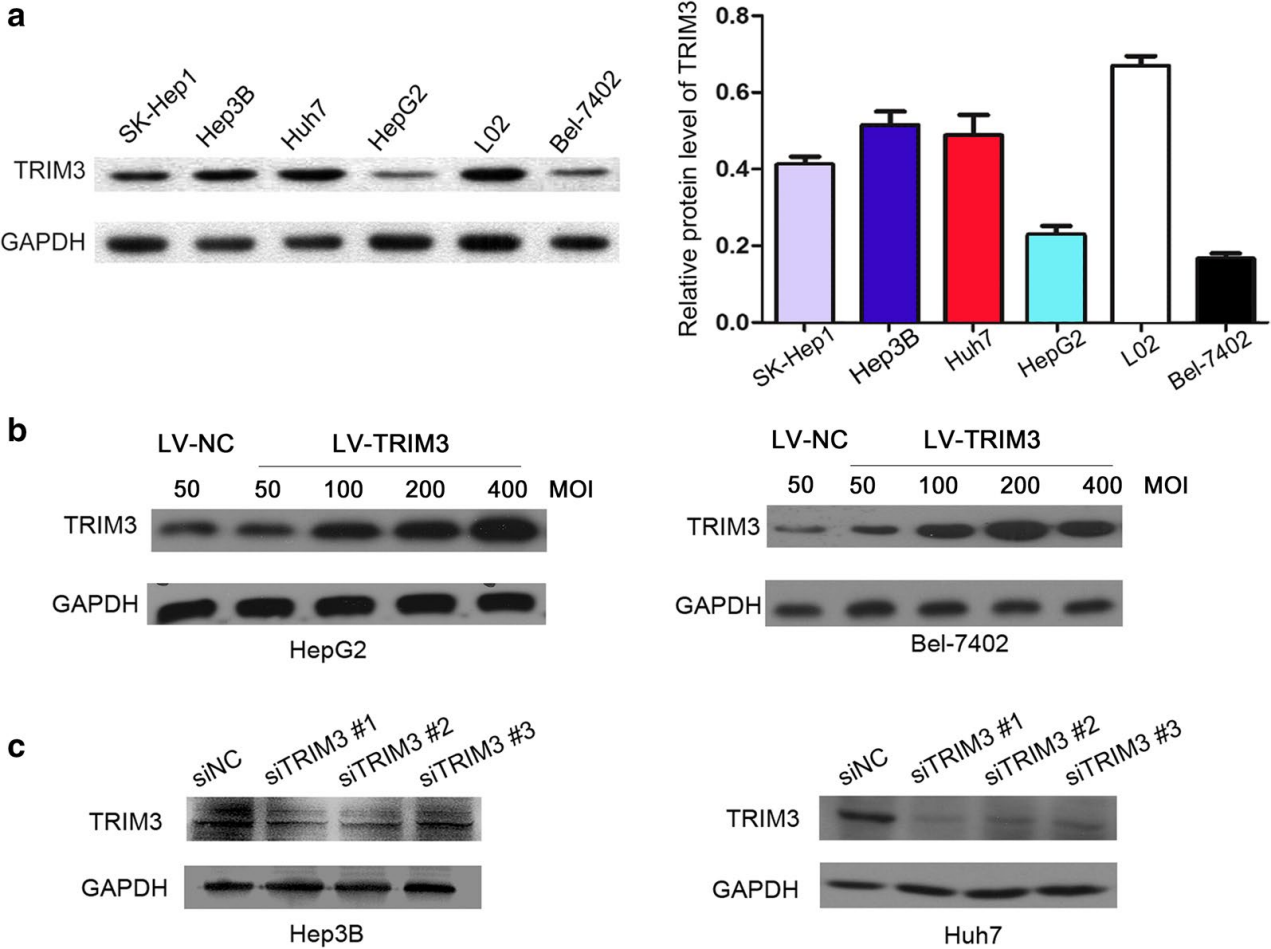
The expression of tripartite motif-containing 3 (TRIM3) protein in liver cancer cell lines evaluated with Western blotting. **a** Compared with the normal liver cell line L02, Bel-7402 and HepG2 cell lines have much lower levels of TRIM3. Relative protein levels of TRIM3 in different cell lines are shown as mean ± standard deviation (SD). Glyceraldehyde 3-phosphate dehydrogenase (GAPDH), was used as internal reference. **b** TRIM3 expression in HepG2 and Bel-7402 cells infected with lentiviruses expressing TRIM3 (LV-TRIM3) at multiplicities of infection (MOIs) of 50, 100, 200, and 400 was higher than that in cells infected with negative control vector (LV-NC, MOI of 50) on day 16 after infection. HepG2 and Bel-7402 cells infected with lentiviruses at an MOI of 200 were selected to be used in function assays. **c** Among the three TRIM3-targeted small interfering RNAs (siTRIM3), siTRIM3 #1 and siTRIM3 #2 showed higher knockdown efficiencies after 48-h transfection. siNC, negative control small interfering RNAs

We estimated the protein expression levels of TRIM3 in five liver cancer cell lines by Western blotting. As shown in Fig. 1a, compared with the normal liver cell line L02, five liver cancer cell lines (SK-Hep1, Hep3B, Huh7, HepG2, and Bel-7402), especially Bel-7402 and HepG2, had lower levels of TRIM3. Based on the TRIM3 expression levels, Hep3B, Huh7, HepG2, and Bel-7402 cell lines were selected for subsequent experiments.

### Overexpression and down-regulation of TRIM3 expression in liver cancer cell lines

HepG2 and Bel-7402 cells with relatively low TRIM3 expression were infected with LV-TRIM3 and LV-NC at different MOIs. TRIM3 expression in HepG2/TRIM3 and Bel-7402/TRIM3 cells was confirmed by Western blotting (Fig. 1b). The cells infected with lentiviruses at an MOI of 200 were selected and used in subsequent experiments. Huh7 and Hep3B cells with relatively high TRIM3 expression were transfected with siTRIM3 #1, siTRIM3 #2, and siTRIM3 #3. The knockdown efficiency of TRIM3 was evaluated with Western blotting after 48-h transfection. TRIM3 expression levels were markedly decreased in both cell lines transfected with either siTRIM3 #1 or siTRIM3 #2 (Fig. 1c).

### TRIM3 inhibited the growth of liver cancer cells

**Fig. 2:**
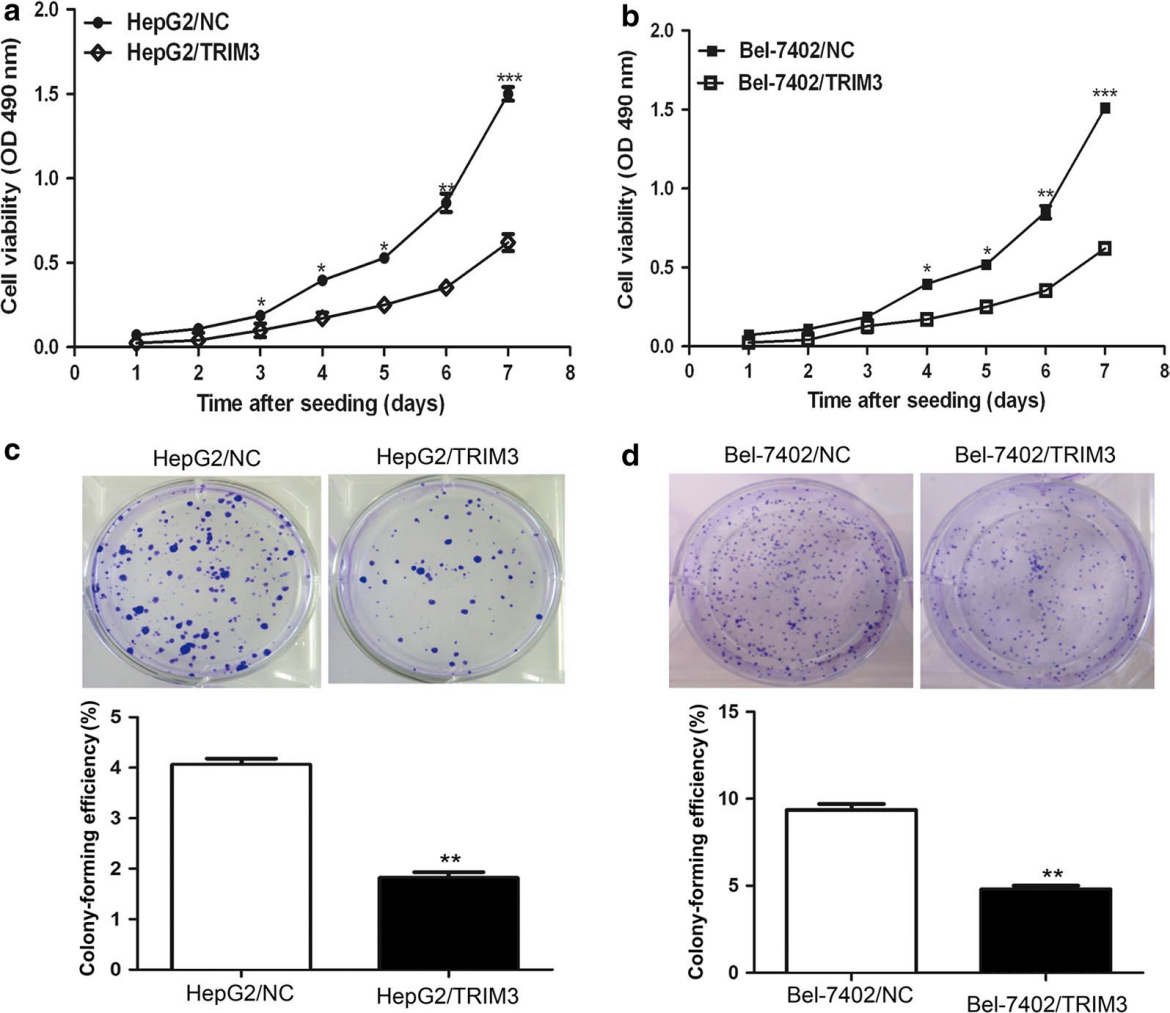
Inhibitory effect of TRIM3 on the growth of HepG2 and Bel-7402 cells. **a**, **b** Cell proliferation assay results show that TRIM3 overexpression suppressed the proliferation of HepG2 (**a**) and Bel-7402 (**b**) cells. OD, optical density. **c**, **d** *Top* representative images of decreased colony formation of HepG2/TRIM3 and Bel-7402/TRIM3 cells in a monolayer culture. *Bottom* colony-forming efficiencies were calculated after 12-day conventional culture. Measurements were carried out in triplicate, and experiments were repeated three times. Data are presented as mean ± SD. *P* values were calculated using Student’s *t* test. *P < 0.05, **P < 0.01, and ***P < 0.001, versus cells transfected with LV-NC

**Fig. 3:**
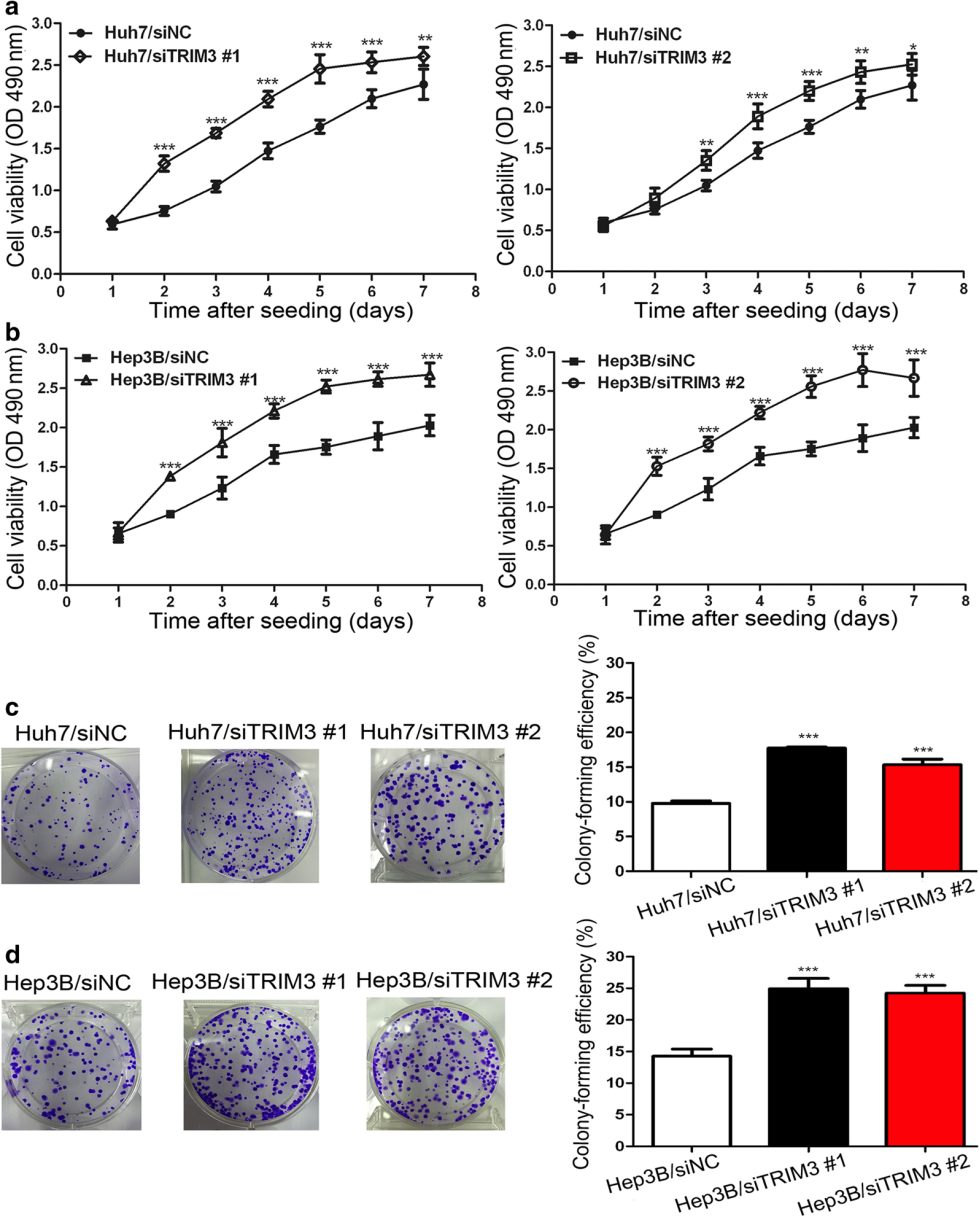
Promotion of Huh7 and Hep3B cell growth by TRIM3 silencing. **a**, **b** Proliferation assay results show that silencing TRIM3 expression promoted proliferation of Huh7 (**a**) and Hep3B (**b**) cells. **c**, **d** *Left* representative images of increased colony formation in a monolayer culture by silencing TRIM3. *Right* colony-forming efficiencies were calculated after 12-day conventional culture. Measurements were carried out in triplicate, and experiments were repeated three times. Data are presented as mean ± SD. *P* values were calculated using Student’s *t* test. *P < 0.05, **P < 0.01, and ***P < 0.001, versus cells transfected with siNC

To evaluate the role of TRIM3 in the growth of liver cancer cells, cell proliferation and colony formation assays were conducted. Cell proliferation assay results showed that starting from the 4th day, the proliferation of HepG2/TRIM3 and Bel-7402/TRIM3 cells was significantly lower than that of HepG2/NC and Bel-7402/NC cells (both *P* < 0.05, Fig. 2a, b). Colony formation assay results revealed that, compared with HepG2/NC and Bel-7402/NC cells, HepG2/TRIM3 and Bel-7402/TRIM3 cells showed approximately 50% reduction in colony formation after 12-day conventional culture (both *P* < 0.01, Fig. 2c, d). Whereas, Huh7/siTRIM3 and Hep3B/siTRIM3 cells showed enhanced proliferation (all *P* < 0.05, Fig. 3a, b) and colony-forming abilities compared with Huh7/siNC and Hep3B/siNC cells (all *P* < 0.001, Fig. 3c, d). These data indicate that TRIM3 may inhibit the growth of liver cancer cells.

### TRIM3 induced G_0_/G_1_ phase arrest in liver cancer cells

**Fig. 4:**
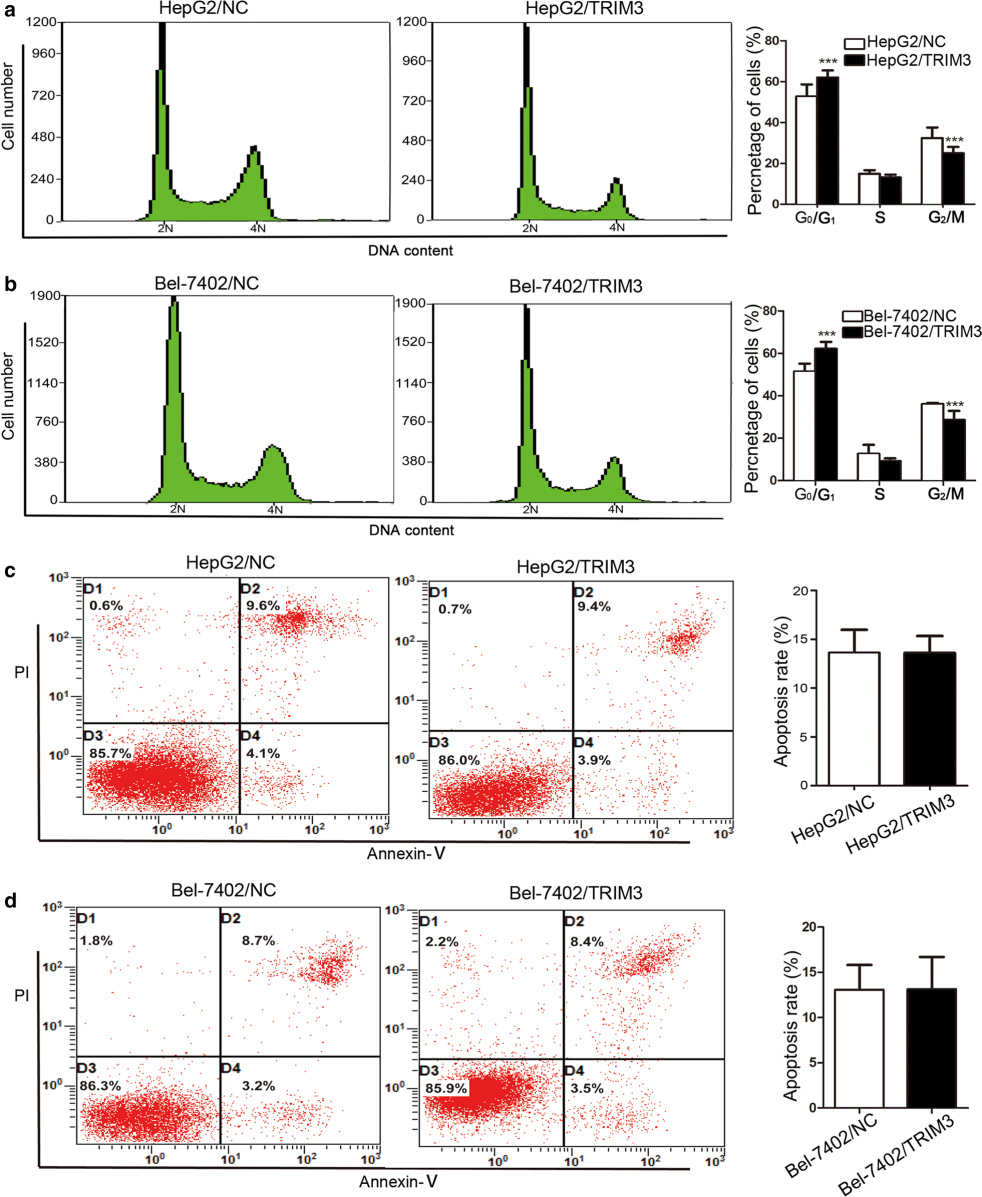
Effects of TRIM3 overexpression on the cell cycle and apoptosis of HepG2 and Bel-7402 cells. **a**, **b** Effect of TRIM3 overexpression on the cell cycle was determined using flow cytometry. TRIM3 overexpression caused G_0_/G_1_ phase arrest in HepG2 (**a**) and Bel-7402 (**b**) cells. **c**, **d** Effect of TRIM3 overexpression on apoptosis of HepG2 and Bel-7402 cells was determined with Annexin V-propidium iodide (PI) staining method. Apoptosis rates are not significantly different between HepG2 (**c**) and Bel-7402 (**d**) cells infected with LV-TRIM3 and those infected with LV-NC. *P* values were calculated using Student’s *t* test. *P < 0.05, **P < 0.01, and ***P < 0.001, versus cells infected with LV-NC

**Fig. 5:**
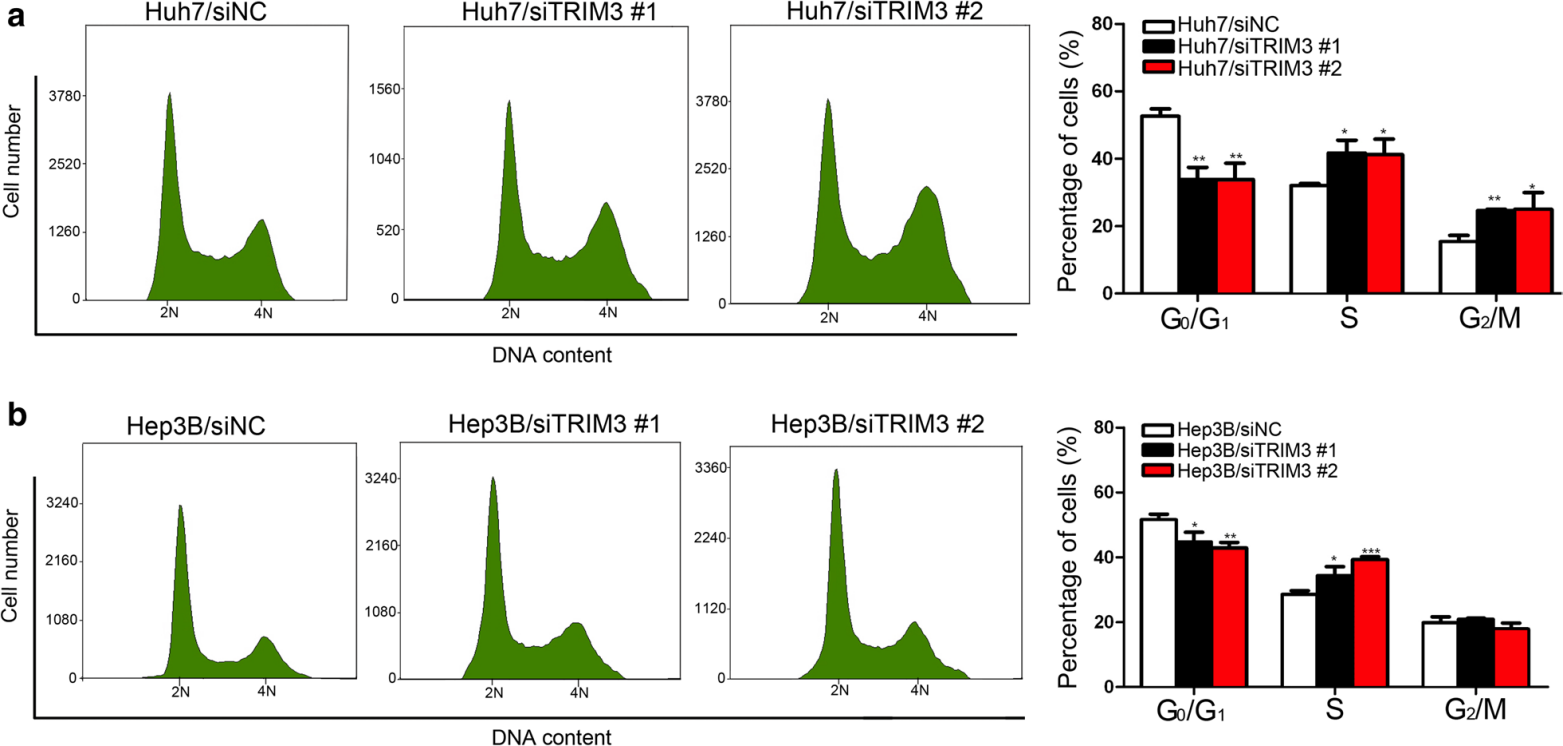
Effects of TRIM3 silencing on the cell cycle of Huh7 and Hep3B cells. The cells were collected for cell cycle distribution analysis with flow cytometry after they were transfected with siTRIM3 or siNC for 48 h. The percentages of cells in the G_0_/G_1_ phase were significantly decreased in Huh7 (**a**) and Hep3B (**b**) cells transfected with siTRIM3. *P* values were calculated using Student’s *t* test. *P < 0.05, **P < 0.01, and ***P < 0.001, versus cells transfected with siNC

To study the potential mechanism underlying the anti-proliferation effect of TRIM3, we conducted cell cycle and apoptosis analyses using flow cytometry. Cell cycle analyses showed that compared with HepG2/NC and Bel-7402/NC cells, the HepG2/TRIM3 and Bel-7402/TRIM3 cells had significantly increased percentages in G_0_/G_1_ phase and notably decreased percentages in G_2_/M phase (all *P*  <  0.001, Fig. 4a, b). Contrarily, knockdown of TRIM3 in Huh7 and Hep3B cells led to significantly decreased percentages in the G_0_/G_1_ phase (all *P* <  0.05, Fig. 5). No significant differences in the number of apoptotic cells were observed between TRIM3-overexpressing HepG2 and Bel-7402 cells and those infected with LV-NC (both *P* > 0.05, Fig. 4c, d). These results suggest that TRIM3 may arrest cell cycle progression in the G_0_/G_1_ phase and thus inhibit cell proliferation.

### TRIM3 suppressed the migration and invasion of liver cancer cells

**Fig. 6:**
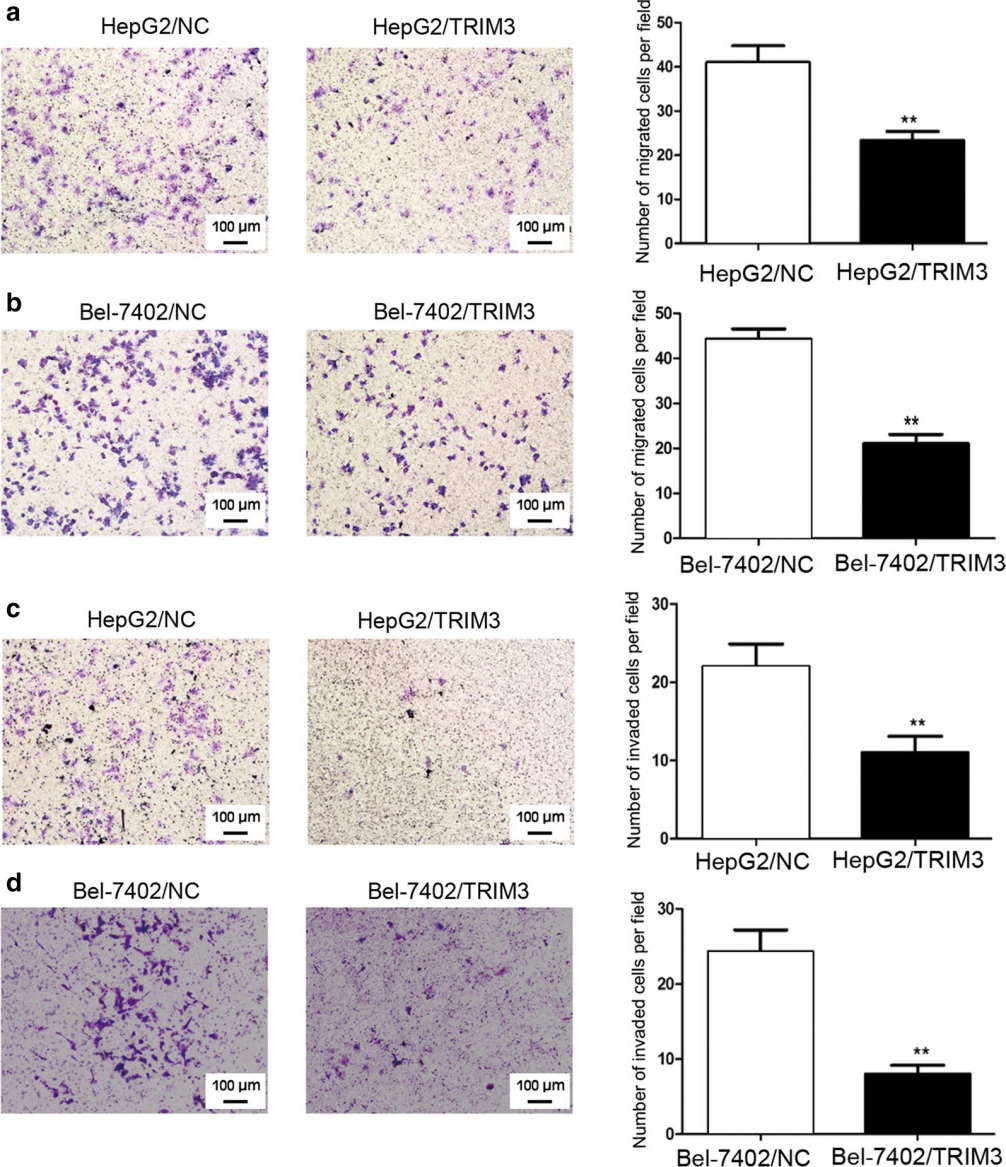
Overexpression of TRIM3 suppressed migration and invasion of HepG2 and Bel-7402 cells. Representative images of cell invasion and migration are shown on the *left* (×100 magnification), and quantification of migrated or invaded cells in 10 randomly selected fields are shown on the *right*. **a**, **b** TRIM3 overexpression inhibited migration of HepG2 (**a**) and Bel-7402 (**b**) cells. **c**, **d** TRIM3 overexpression significantly attenuated invasion of HepG2 (**c**) and Bel-7402 (**d**) cells. Data are shown as mean  ±  SD of three independent experiments. *P* values were calculated using Student’s *t*-test. *P < 0.05, **P < 0.01, and ***P < 0.001, versus cell transfected with LV-NC

**Fig. 7:**
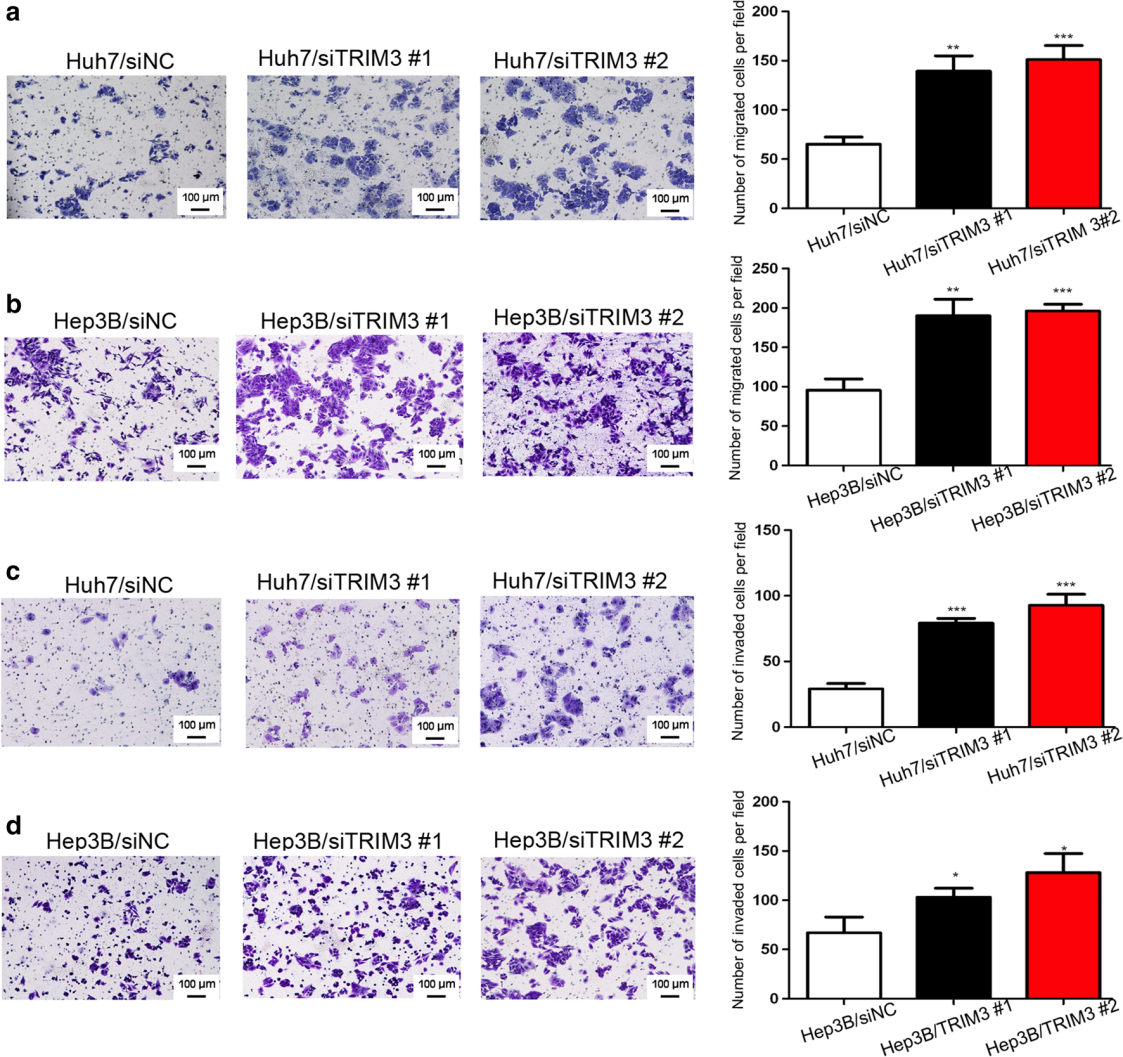
Silencing TRIM3 promoted migration and invasion of Huh7 and Hep3B cells. Representative images of cell invasion and migration are shown on the* left* (×100 magnification), and quantification of migrated or invaded cells in 10 randomly selected fields are shown on the* right*. **a**, **b** Silencing TRIM3 promoted migration of Huh7 (**a**) and Hep3B (**b**) cells. **c**, **d** Silencing TRIM3 promoted invasion of Huh7 (**c**) and Hep3B (**d**) cells. Data are shown as mean  ±  SD of three independent experiments. *P* values were calculated using Student’s *t* test. *P < 0.05, **P < 0.01, and ***P < 0.001, versus cells transfected with siNC

We studied the effects of TRIM3 on migration and invasion of liver cancer cells using transwell assays with or without Matrigel, respectively. Transwell assay results showed that overexpression of TRIM3 in HepG2 and Bel-7402 cells led to approximately 50% reduction in the numbers of migrated and invaded cells compared with infection of LV-NC (all *P* < 0.01, Fig. 6). However, the migration and invasion abilities of Huh7 and Hep3B cells transiently transfected with siTRIM3 was significantly increased compared with those of cells transfected with siNC (all *P* < 0.05, Fig. 7). These results suggest that TRIM3 can efficiently repress the motility and invasiveness of liver cancer cells.

### TRIM3 suppressed the growth and metastasis of HepG2 and Bel-7402 cells in nude mice

**Fig. 8:**
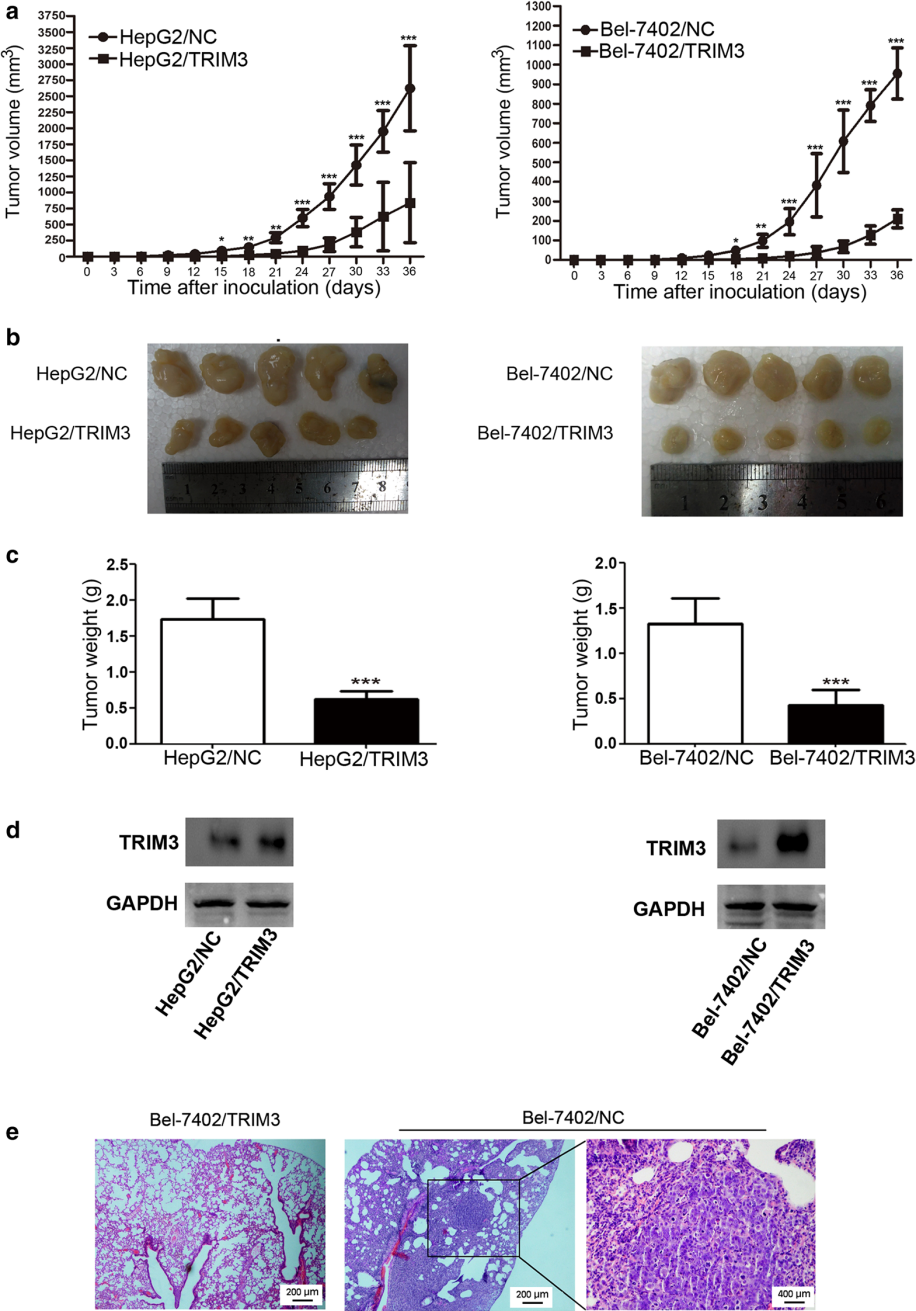
Overexpression of TRIM3 suppressed the growth and metastasis of HepG2 and Bel-7402 cells in vivo. HepG2 or Bel-7402 cells infected with LV-NC and LV-TRIM3 were injected into nude mice. Tumor volumes were measured every 3 days. At the end of the experiment, the mice were killed, and the tumors were harvested for the volume and weight measurement. **a** Tumor growth rates are reduced in mice injected with TRIM3-overexpressing cells. **b** Photographs of dissected tumors from nude mice. Final tumor sizes are smaller in mice injected with TRIM3-overexpressing cells than those in mice injected with LV-NC-infecting cells. **c** The final tumor weights are reduced in mice injected with TRIM3-overexpressing cells. **d** TRIM3 protein expression is higher in tumors in mice injected with TRIM3-overexpressing cells than in those in mice injected with LV-NC-infecting cells. Data are presented as mean ± SD. *P* values were calculated using Student’s *t* test. *P < 0.05, **P < 0.01, and ***P < 0.001, versus cells infected with LV-NC. **e** Overexpression of TRIM3 inhibited lung metastasis in vivo. Representative histological images of lung sections with hematoxylin and eosin (HE) staining are shown. *Black frame* indicates the metastatic nodule

To determine the effect of TRIM3 on tumor growth in vivo, HepG2 and Bel-7402 cells infected with LV-TRIM3 or LV-NC were subcutaneously injected into nude mice, respectively. Tumors were successfully formed in all mice. As shown in Fig. 8a, b, the mean tumor volumes in mice injected with TRIM3-overexpressing cells were significantly smaller than those in mice injected with LV-NC-infecting cells (746 mm^3^ vs. 2128 mm^3^ for HepG2; 308 mm^3^ vs. 1286 mm^3^ for Bel-7402; both *P* < 0.001). The mean tumor weights in mice injected with TRIM3-overexpressing cells were also markedly lower than those in mice injected with LV-NC-infecting cells (0.8 g vs. 1.8 g for HepG2; 0.5 g vs. 1.3 g for Bel-7402; both *P* < 0.001, Fig. 8c). Then, we examined TRIM3 expression in the tumor samples with Western blotting. We observed that TRIM3 expression in the tumors formed with TRIM3-overexpressing cells was higher than that formed with LV-NC-infecting cells (Fig. 8d). To determine the effect of TRIM3 on metastasis in vivo, Bel-7402/TRIM3 cells and Bel-7402/NC cells were injected into nude mice through the tail veins. We found that two of the mice injected with Bel-7402/NC cells developed lung metastases, but none of the mice injected with Bel-7402/TRIM3 cells developed lung metastases (Fig. 8e).

## Discussion

In the present study, we found that TRIM3 expression was lower in liver cancer cells than in normal liver cells. When TRIM3 was overexpressed, cell proliferation, colony formation, migration, and invasion were inhibited and cells were arrested in G_0_/G_1_ phase. When TRIM3 expression was knocked down, the above functions and cell percentages in S and G_2_/M phase were increased. Furthermore, in vivo tumor growth and lung metastasis were repressed when TRIM3 was overexpressed.

In our previous study [25], we found that *TRIM3* expression was significantly down-regulated in most HCC tumor tissues, compared with corresponding noncancerous tissues. We also found that low *TRIM3* expression was an independent indicator of poor prognosis in HCC patients. In the present study, we found that TRIM3 overexpression significantly inhibited cell proliferation and colony formation, whereas TRIM3 silencing promoted these processes. The role of TRIM3 in inhibiting tumor growth was also observed in vivo. TRIM3 overexpression in HepG2 and Bel-7402 cells significantly delayed tumor growth in mice. These results were in line with our clinical findings that the low level of TRIM3 was significantly associated with large tumors [25]. Consistent with our results, previous studies also demonstrated that TRIM3 overexpression inhibited glioma cell proliferation and tumor growth [27, 28]. Additionally, reducing TRIM3 expression promoted tumor development and accelerated the progression of oligodendroglioma in a mouse model [29].

Our functional experiments also showed that TRIM3 overexpression in HepG2 and Bel-7402 cells induced G_0_/G_1_ phase arrest and reduced the percentage of cells in the G_2_/M phase. This result indicated that TRIM3 may suppress tumorigenicity by inducing cell cycle arrest. A similar cell cycle arrest in glioblastoma cells was reported previously [28]. TRIM3-induced cell cycle arrest was associated with its function of sequestering and preventing P21 from facilitating the accumulation of cyclinD1-Cdk4 [29]. However, whether TRIM3 acts on cell cycle-related proteins in liver cancer needs to be determined in future studies. Cell apoptosis analyses showed that TRIM3 overexpression did not significantly affect apoptosis, suggesting that TRIM3 may not play a role in the apoptosis of liver cancer cells.

Our study also showed that TRIM3 overexpression inhibited the migration and invasion of liver cancer cells. Contrarily, silencing TRIM3 promoted cell motility. These findings were consistent with the results of our clinicopathologic analysis, which showed that low *TRIM3* expression was significantly associated with advanced tumor stage [25]. Furthermore, we performed in vivo metastasis assay in nude mice to investigate whether TRIM3 overexpression could alter the in vivo metastatic ability of liver cancer cells. We found that mice inoculated with TRIM3-overexpressing cells did not have metastatic lung nodules whereas those inoculated with LV-NC-infecting cells did have. However, the precise molecular mechanism by which TRIM3 suppresses liver cancer cell metastasis remains uncertain, whether TRIM3 acts on cell mobility-related proteins in liver cancer needs to be determined in future studies.

In summary, our results demonstrated that TRIM3 suppressed the tumorigenicity of liver cancer cells by inhibiting proliferation, colony formation, migration, and invasion and by inducing cell cycle arrest of tumor cells. The mouse model experiment further showed that TRIM3 overexpression significantly inhibited tumor growth and lung metastasis. Taken together, although the precise molecular mechanism by which TRIM3 regulates liver cancer progression remains unknown, our results suggest that TRIM3 may serve as a tumor suppressor in liver cancer.

## References

[1] Forner A, Llovet JM, Bruix J (2012). Hepatocellular carcinoma. Lancet.

[2] Miki D, Ochi H, Hayes CN, Aikata H, Chayama K (2012). Hepatocellular carcinoma: towards personalized medicine. Cancer Sci.

[3] Chen JL, Lin XJ, Zhou Q, Shi M, Li SP, Lao XM (2016). Association of HBV DNA replication with antiviral treatment outcomes in the patients with early-stage HBV-related hepatocellular carcinoma undergoing curative resection. Chin J Cancer..

[4] Hsu CY, Liu PH, Hsia CY, Lee YH, Nagaria TS, Lee RC (2016). Surgical resection is better than transarterial chemoembolization for patients with hepatocellular carcinoma beyond the milan criteria: a prognostic nomogram study. Ann Surg Oncol.

[5] Toshikuni N, Tsutsumi M, Takuma Y, Arisawa T (2014). Real-time Image fusion for successful percutaneous radiofrequency ablation of hepatocellular carcinoma. J Ultrasound Med.

[6] Poon RT, Fan ST (2004). Hepatectomy for hepatocellular carcinoma: patient selection and postoperative outcome. Liver Transpl.

[7] Zhang J, Zhou ZG, Huang ZX, Yang KL, Chen JC, Chen JB (2016). Prospective, single-center cohort study analyzing the efficacy of complete laparoscopic resection on recurrent hepatocellular carcinoma. Chin J Cancer.

[8] Ng KK, Lo CM, Chan SC, Chok KS, Cheung TT, Fan ST (2010). Liver transplantation for hepatocellular carcinoma: the Hong Kong experience. J Hepatobiliary Pancreat Sci.

[9] Qi X, Ng KT, Lian Q-Z (2014). Clinical significance and therapeutic value of glutathione peroxidase 3 (GPx3) in hepatocellular carcinoma. Oncotarget.

[10] Villanueva A, Hoshida Y (2011). Depicting the role of TP53 in hepatocellular carcinoma progression. J Hepatol.

[11] Hannah J, Zhou PB (2013). The CUL4A ubiquitin ligase is a potential therapeutic target in skin cancer and other malignancies. Chin J Cancer.

[12] Nisole S, Stoye JP, Saib A (2005). TRIM family proteins: retroviral restriction and antiviral defence. Nat Rev Microbiol.

[13] Ozato K, Shin DM, Chang TH, Morse HC (2008). TRIM family proteins and their emerging roles in innate immunity. Nat Rev Immunol.

[14] Short KM, Hopwood B, Yi Z, Cox TC (2002). MID1 and MID2 homo- and heterodimerise to tether the rapamycin-sensitive PP2A regulatory subunit, alpha 4, to microtubules: implications for the clinical variability of X-linked Opitz GBBB syndrome and other developmental disorders. BMC Cell Biol.

[15] Niikura T, Hashimoto Y, Tajima H, Ishizaka M, Yamagishi Y, Kawasumi M (2003). A tripartite motif protein TRIM11 binds and destabilizes Humanin, a neuroprotective peptide against Alzheimer’s disease-relevant insults. Eur J Neurosci.

[16] Takahata M, Bohgaki M, Tsukiyama T, Kondo T, Asaka M, Hatakeyama S (2008). Ro52 functionally interacts with IgG1 and regulates its quality control via the ERAD system. Mol Immunol.

[17] Kedar V, McDonough H, Arya R, Li HH, Rockman HA, Patterson C (2004). Muscle-specific RING finger 1 is a bona fide ubiquitin ligase that degrades cardiac troponin I. Proc Natl Acad Sci USA.

[18] Bodine SC, Latres E, Baumhueter S, Lai VK, Nunez L, Clarke BA (2001). Identification of ubiquitin ligases required for skeletal muscle atrophy. Science.

[19] Gatt ME, Takada K, Mani M, Lerner M, Pick M, Hideshima T (2013). TRIM13 (RFP2) downregulation decreases tumour cell growth in multiple myeloma through inhibition of NF Kappa B pathway and proteasome activity. Br J Haematol.

[20] Cambiaghi V, Giuliani V, Lombardi S, Marinelli C, Toffalorio F, Pelicci PG (2012). TRIM proteins in cancer. Adv Exp Med Biol.

[21] Appikonda S, Thakkar KN, Barton MC (2016). Regulation of gene expression in human cancers by TRIM24. Drug Discov Today Technol.

[22] Herquel B, Ouararhni K, Khetchoumian K, Ignat M, Teletin M, Mark M (2011). Transcription cofactors TRIM24, TRIM28, and TRIM33 associate to form regulatory complexes that suppress murine hepatocellular carcinoma. Proc Natl Acad Sci USA.

[23] Koi M, Johnson LA, Kalikin LM, Little PF, Nakamura Y, Feinberg AP (1993). Tumor cell growth arrest caused by subchromosomal transferable DNA fragments from chromosome 11. Science.

[24] El-Husseini AE, Fretier P, Vincent SR (2000). Cloning and characterization of a gene (RFN22) encoding a novel brain expressed ring finger protein (BERP) that maps to human chromosome 11p15.5. Genomics.

[25] Chao J, Zhang XF, Pan QZ, Zhao JJ, Jiang SS, Wang Y (2014). Decreased expression of TRIM3 is associated with poor prognosis in patients with primary hepatocellular carcinoma. Med Oncol.

[26] Pan QZ, Pan K, Weng DS, Zhao JJ, Zhang XF, Wang DD (2015). Annexin A3 promotes tumorigenesis and resistance to chemotherapy in hepatocellular carcinoma. Mol Carcinog.

[27] Raheja R, Liu Y, Hukkelhoven E, Yeh N, Koff A (2014). The ability of TRIM3 to induce growth arrest depends on RING-dependent E3 ligase activity. Biochem J.

[28] Chen G, Kong J, Tucker-Burden C, Anand M, Rong Y, Rahman F (2014). Human Brat ortholog TRIM3 is a tumor suppressor that regulates asymmetric cell division in glioblastoma. Cancer Res.

[29] Liu YH, Raheja R, Yeh N, Ciznadija D, Pedraza AM, Ozawa T (2014). TRIM3, a tumor suppressor linked to regulation of p21(Waf1/Cip1). Oncogene.

